# Airborne transmission of COVID-19 and the role of face mask to prevent it: a systematic review and meta-analysis

**DOI:** 10.1186/s40001-020-00475-6

**Published:** 2021-01-02

**Authors:** Seyed-Amir Tabatabaeizadeh

**Affiliations:** Nutrition and Biochemistry Department, School of Medicine, Social Development and Health Promotion Research Center, Gonabad University of Medical Sciences, Gonabad, Iran

**Keywords:** SARS-CoV-2, COVID-19, Transmission, Masks, Meta-analysis

## Abstract

**Background and aims:**

Severe acute respiratory syndrome coronavirus 2 (SARS-CoV-2), belonging to the Coronaviridae family, is agent of 2019 novel coronavirus disease (COVID-19). COVID-19 emerged in Wuhan, Hubei province of China, in early December 2019 and is now considered a pandemic. This study aimed to investigate the airborne transmission of COVID-19 and the role of face mask to prevent it.

**Methods:**

A systematic search for English-language literature was done via PUBMED/Medline and Google Scholar up to October 2020. There was two search strategy; for airborne transmission and the role of face mask for prevention of SARS-CoV-2 infection. Based on a fixed and random effects model, the RR and 95% CI were used to evaluate the combined risk. This meta-analysis followed Preferred Reporting Items for Systematic Reviews and Meta-analysis (PRISMA) Guidelines.

**Results:**

After eligibility assessment, four articles with a total of 7688 participants were included in this meta-analysis. The result of this meta-analysis has shown significant reduction in infection with face mask use; the pooled RR (95%CI) was 0.12 [0.06, 0.27] (*P* < 0.001).

**Conclusion:**

In conclusion, this meta-analysis suggests that there is association between face mask use and reduction of COVID-19. However, COVID-19 spreads primarily with contact routes and respiratory droplets, but its transmissibility has many mysteries yet and there is controversy about airborne transmission of COVID-19.

## Introduction

Severe acute respiratory syndrome coronavirus 2 (SARS-CoV-2) is a member of the Coronaviridae family, and its RNA genome size is 29,891 nucleotides and encoding 9860 amino acids [1]. SARS-CoV-2 is agent of 2019 novel coronavirus disease (COVID-19) and spreads primarily with contact routes and respiratory droplets. COVID-19 emerged in Wuhan, Hubei province of China, in early December 2019 and is now considered a pandemic [2]. More than 37.1 million infected cases are confirmed in more than 180 countries, including 1.07 million deaths (as of October 11, 2020).

**Fig. 1:**
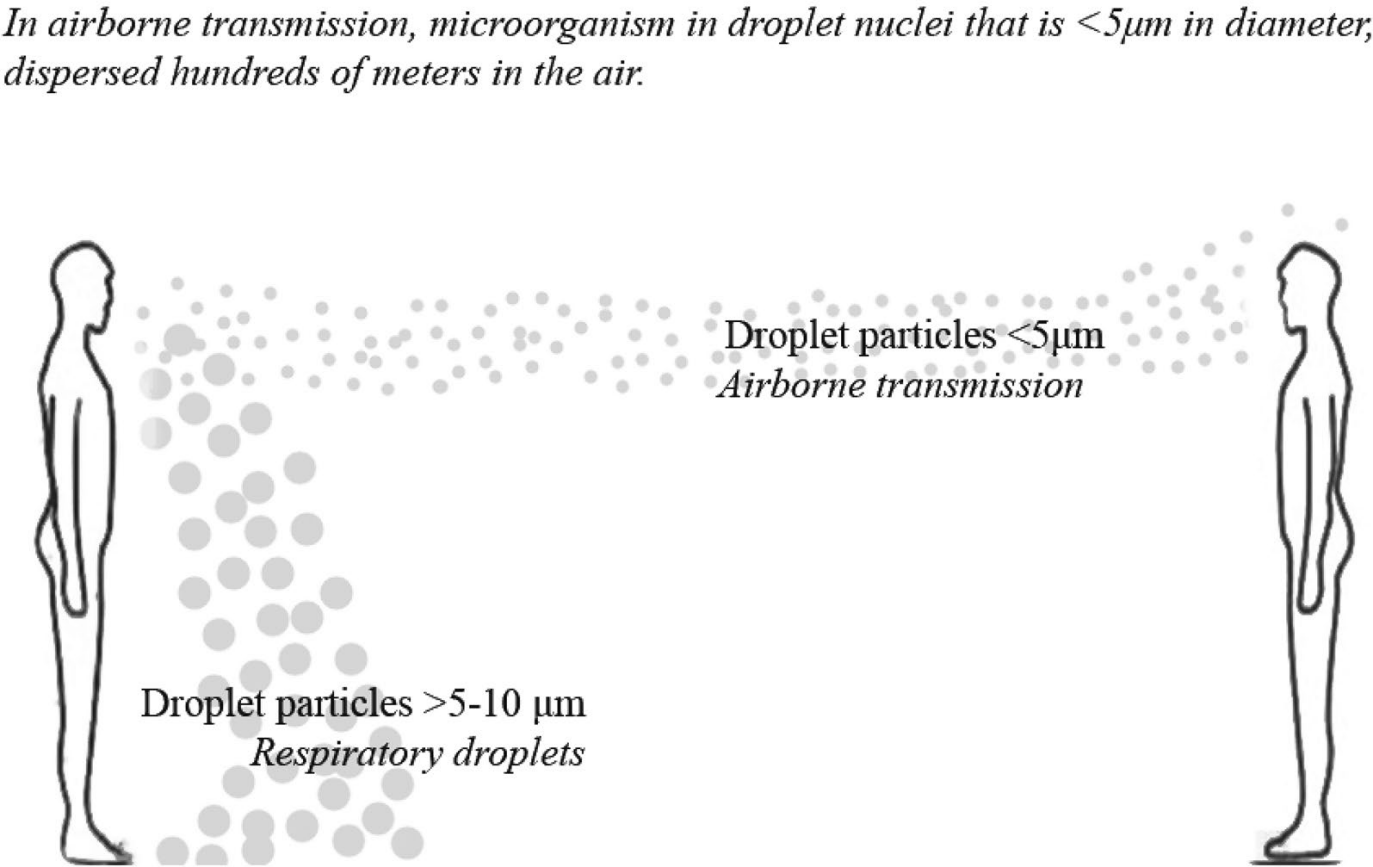
Airborne transmission

Respiratory droplets are referred to droplet particles > 5–10 μm in diameter [3]. As mentioned above, COVID-19 spreads primarily with respiratory droplets and there is controversy about airborne transmission (droplet particles < 5 μm in diameter or droplet nuclei) (Fig. 1).

Aerosols are liquid particles dispersed in the air and contains particles, like microorganism or industrial particles. It is hypothesizing that when an infected person with SARS-CoV-2 breathes heavily, sneezes, or coughs, the SARS-CoV-2 will be excreted and made bio-aerosols. As mentioned above, bio-aerosols < 5 μm in diameter causes airborne transmission; however, larger ones put down on surfaces.

Droplet transmission caused when a person’s saliva discharged by coughing or sneezing and occurs in a space of 1 m (close contact). In this situation, there is risk of having conjunctival or mucosal infection by infective droplets. Furthermore, droplet transmission occurs through contact routes in the close environment of the infected person [4]. In contrast, in airborne transmission, microorganism in droplet nuclei, that is, < 5 μm in diameter, is dispersed hundreds of meters in the air and can remain for a long time.

For reduction in infection among the persons who are in contact with infected people, there is advice about physical distancing. Face masks is a debating option in media and public health advisors especially for general population [5]. Face masks were used for decades for prevention of viral infections especially for health cares, but lack of personal protective equipment (PPE) for health care providers makes it difficult for general recommendation [6].

Previous studies have shown that face mask use could be effective for reducing community spread of the 2003 SARS infection [7, 8]. Rengasamy et al. have shown that cloth masks (70% cotton and 30% polyester) and 100% cotton masks can filtrate 40–60% of NaCl aerosols with 75 nm count median diameter at 5.5 cm/s face velocity [9]. Furthermore, Kin-Fai Ho et al. have shown that cotton masks and medical masks have no significant difference in cough counts when they are used in the car or room [10]. Meta-analysis has shown that medical masks have protective effects against respiratory infection [11]; however, there are controversial results in clinical trials [12–14]. Recommendation for using face masks or physical distancing needs to be based on clinical evidence. But there is little evidence about the COVID-19 and previous meta-analysis mostly did not consider face masks for SARS-CoV-2 infection and evaluate its effect on prevention from other viral infections, like MERS and SARS. Also, there is controversy about airborne transmission of COVID-19 and close contact, and respiratory droplets do not explain all infections. Therefore, this study aimed to meta-analyze by combining related studies and analyze the pooled RR of face mask use and COVID-19 in asymptomatic individuals without COVID-19 infection and confirmed COVID-19 patients. Also, evaluate airborne transmission of SARS-CoV-2.

## Materials and methods

This meta-analysis followed Preferred Reporting Items for Systematic Reviews and Meta-analysis (PRISMA) Guidelines for evaluation of the role of face mask in prevention of COVID-19 [15].

### Population, Interventions, Comparators, Outcomes, and Study Designs (PICOS)

Population: Asymptomatic individuals without COVID-19 infection and confirmed COVID-19 patients.

Intervention: To evaluate the role of face mask in prevention of COVID-19.

Comparators: Effect of the face mask in risk of transmission of COVID-19.

Outcomes: Risk of COVID-19 infection.

Study designs: A systematic review and meta-analysis.

Also, there was a separate search strategy to evaluate airborne transmission of COVID-19. The author has no source of funding to report.

A systematic search for English-language literature was done via PUBMED/Medline (Medical Literature Analyses and Retrieval System Online) and Google Scholar (Cochrane guideline suggested it as the gray literature) up to October 2020.

Search terms included SARS-CoV-2, COVID-19, transmission, and masks. In addition, publications that were not recognized in two databases were identified from review articles and reference lists of included papers. Conference proceeding and abstracts were excluded. Data extraction has done with piloted forms.

Studies were included in this meta-analysis if RR (95% CI) for association of the face mask use with COVID-19 could be obtained. Studies risk of bias was evaluated for meta-analysis by the Newcastle–Ottawa scale [16].

95% confidence interval (95% CI) was considered as effective size in this analysis. For assessing heterogeneity, *I*^2^ and Chi-square tests were done. *I*^2^ was categorized as low (0–50%), moderate (51–75%), or high (> 75%) for assess heterogeneity. P < 0.05 was considered as statistically significant and tests were two tailed. Funnel plots and Egger regression asymmetry analysis were used for evaluation of publication bias [17]. Stata 14.0 (StataCorp, College Station, TX, USA) was used for data analysis.

## Results

### General characteristics of studies

**Fig. 2:**
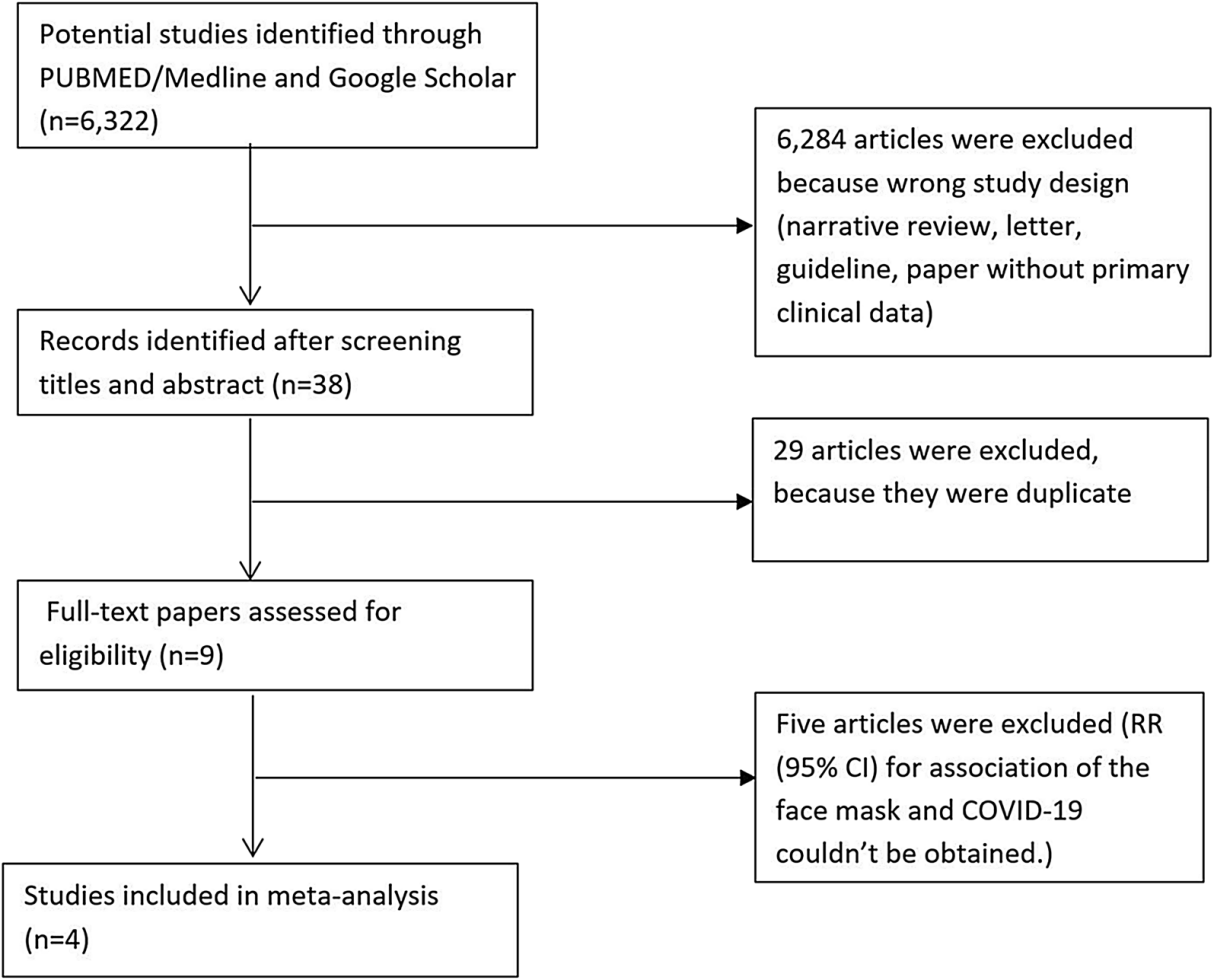
Flow chart of literature search for meta-analysis. RR, relative risk

The general characteristics of studies that were included in this meta-analysis are shown in Table 1. After eligibility assessment, four articles with a total of 7688 participants were included in this meta-analysis [18–21]. Flow chart of literature search is shown in Fig. 2.

**Table 1:** Studies showing the association of face mask use with COVID-19 infection

Study (year)	Country	Sample size (*n*)	Main findings	RR (95% CI)
Heinzerling et al. [19]	USA	37	A strong association of proximity of the exposed individual without face mask with the risk of infection	0.03 (0.002–0.54)
Doung-ngern et al. [18]	Thailand	1716	All time wearing face masks associated with lower risk of SARS-CoV-2 infection compared to individuals who do not wearing face masks	0.23 (0.09–0.6)
Wang et al. [21]	China	493	Precise occupational protection is necessary for fighting COVID-19	0.04 (0.002–0.63)
Wang et al. [20]	China	5442	Inadequate protection leads to higher risk of infection in medical staff members	0.03 (0.004–0.19)

*RR* relative risk

### Meta-analysis

**Fig. 3:**
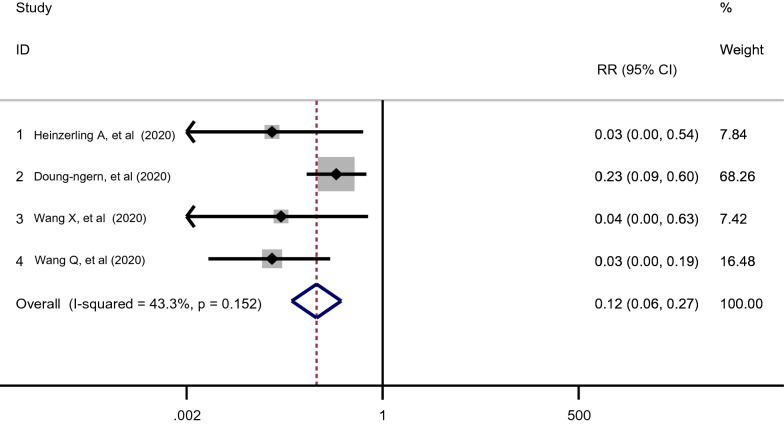
Meta-Analysis showing the association of face mask use with COVID-19 infection. RR, relative risk

The RRs from the four studies and pooled RR are presented in Fig. 3. In total, four studies were used to assess the association between face mask use and SARS-CoV-2 infection. All of them showed that face mask use was linked to a decrease risk of SARS-CoV-2 infection. Overall, meta-analysis of studies suggested that there was a statistically significant association between face mask use and COVID-19; the pooled RR (95%CI) was 0.12 [0.06, 0.27] (*P* < 0.001) (Fig. 3). The *I*^2^ = 43.3% and *P* = 0.152 indicated evidence of minimal heterogeneity and the fixed-effects model was used.

**Fig. 4:**
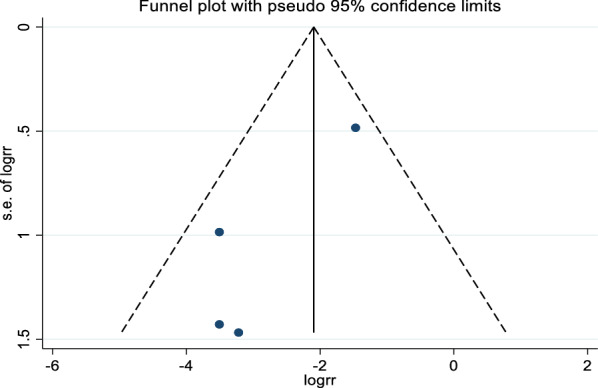
Funnel plot for publication bias

Each study was excluded individually to assess the reliability of the results and sensitivity analysis. There were no significant changes in the pooled RR. The funnel plot indicated symmetrical distribution of all the included studies in the triangle area. The funnel plot of all the included studies is shown in Fig. 4. Egger’s test has shown no publication bias in the included studies (*P* = 0.08).

## Discussion

Meta-analysis of studies has suggested that the use of the face mask was associated with a decrease risk of SARS-CoV-2 infection (*P* < 0.001). Our findings are consistent with the previous evidence from population researches that have shown association between face mask use and decrease risk of viral infection [6]. Researchers have found that severe acute respiratory syndrome-related coronavirus (SARS-CoV) and Middle Eastern respiratory syndrome coronavirus (MERS-CoV) have airborne transmission [22, 23].

In a recent paper in the *New England Journal of Medicine* (*NEJM*), the authors have evaluated the aerosol stability of SARS-CoV-2 as compared with SARS-CoV [24]. They have generated aerosols with using a three-jet Collison nebulizer and fed into a Goldberg drum [24]. The authors have shown that SARS-CoV-2 remain viable for at least 3 h (duration of their experiment). Interestingly, the reduction in infectious titer of SARS-CoV-2 was similar to SARS-CoV. Since, this study have shown that SARS-CoV-2 is viable in aerosols for hours; airborne transmission of COVID-19 is probable. Also, in the 6th Guideline for COVID-19 that has been published by the NHC on February 19, 2020, SARS-CoV-2 can disperse in the air in an unventilated environment [25].

In another recent study, Po Ying Chia and his colleagues have shown that air samples of two COVID-19 patients were PCR positive for SARS-CoV-2 and the respiratory particles detected at sizes 1–4 and > 4 μm in diameter [26]. Both COVID-19 patients were in day 5 of symptoms.

Furthermore, a study that has been done in Singapore has shown positive air samples in isolation COVID-19 rooms. The samples size ranges from the 1–4 and > 4 µm with a viral load of 1.8–3.4 RNA in 1 L of air which suggested airborne transmission of SARS-CoV-2 (RNA viral load have been assessed by clinical cycle threshold (Ct) value) [26]. In another study in distances more than 1.8 m, there was a viral load of 2.5 RNA in 1 L of air [27]. Also, Liu et al. have evaluated the air samples in Wuhan hospitals. Many of these samples were < 2.5 µm and could be considered as aerosols. In this study, they have reported that in 2 and 3 m of COVID-19 patients every hour there was 31 and113 RNA /m2 [28]. Zhen-Dong Guo, et al. have evaluated samples from potentially contaminated objects to assess the surface transmission [29]. The surface of the objects that were frequently touched by patients in the ICU and GW was highly positive for SARS-CoV-2. The highest rates were for computer mice, doorknobs, and trash cans. These findings along airborne transmission can explain familial cluster infections.

Face masks are a debating option in media and public health advisors especially for general population [5]. Using face masks is feasible. Based on this review and meta-analysis, public health policy makers should consider this and the likelihood of airborne transmission of COVID-19. It is rational to recommend using face masks as an acceptable advice for general population, especially for health care workers and people caring for COVID-19 patients.

This study has some strengths: First, it is the meta-analysis that evaluates the association of the face mask use with COVID-19. Previous meta-analysis mostly did not consider face masks for SARS-CoV-2 infection and evaluate its effect on prevention other viral infections, like MERS and SARS. Second, this meta-analysis included 7688 participants that increased statistical power and make the results more reliable. Third, this study has shown that the use of the face mask was associated significantly with a decrease risk of SARS-CoV-2 infection, the decision can be considered for the prevention of COVID-19 as a public health concern. The non-randomized design of the included studies in this meta-analysis is the most important limitation of this study. Geographical segmentation will increase particular confounding in demographics, and it can impact COVID-19 outcome and bias in the studies geographical distribution must be considered [30]. Also, there might be some recall bias in the included studies. Furthermore, we must consider the lack of enough data for COVID-19 pandemic. Globally well-designed studies, like randomized control trials, for evaluating best protective options against SARS-CoV-2 are necessary.

## Conclusions

In conclusion, this meta-analysis suggests that there is association between face mask use and reduction of COVID-19. Based on the recent publications, we must consider the likelihood of airborne transmission of COVID-19. It is rational to recommend using face masks as an acceptable advice for general population. Of course, it needs more experiments to confirm SARS-CoV-2 airborne transmission.

## Data Availability

Not available.
